# Vocal Cord Paralysis and Feeding Difficulties as Early Diagnostic Clues of Congenital Myasthenic Syndrome with Neonatal Onset: A Case Report and Review of Literature

**DOI:** 10.3390/jpm13050798

**Published:** 2023-05-06

**Authors:** Domenico Umberto De Rose, Sara Ronci, Stefano Caoci, Chiara Maddaloni, Daria Diodato, Michela Catteruccia, Fabiana Fattori, Luca Bosco, Stefano Pro, Immacolata Savarese, Iliana Bersani, Franco Randi, Marilena Trozzi, Duino Meucci, Flaminia Calzolari, Guglielmo Salvatori, Agostina Solinas, Andrea Dotta, Francesca Campi

**Affiliations:** 1 Neonatal Intensive Care Unit, Bambino Gesù Children’s Hospital, IRCCS, 00165 Rome, Italy; sararonci91@gmail.com (S.R.); stefano.caoci@opbg.net (S.C.); chiara.maddaloni@opbg.net (C.M.); immacolata.savarese@opbg.net (I.S.); iliana.bersani@opbg.net (I.B.); flaminia.calzolari@opbg.net (F.C.); guglielmo.salvatori@opbg.net (G.S.); andrea.dotta@opbg.net (A.D.); francesca.campi@opbg.net (F.C.); 2 Neuromuscular and Neurodegenerative Disorders Unit, Bambino Gesù Children’s Hospital, IRCCS, 00165 Rome, Italy; daria.diodato@opbg.net (D.D.); michela.catteruccia@opbg.net (M.C.); luca.bosco@opbg.net (L.B.); 3 Laboratory of Medical Genetics, Translational Cytogenomics Research Unit, Bambino Gesù Children Hospital, IRCCS, 00165 Rome, Italy; fabiana.fattori@opbg.net; 4 Department of Science, University Roma Tre, 00146 Rome, Italy; 5 Developmental Neurology Unit, Bambino Gesù Children’s Hospital, IRCCS, 00165 Rome, Italy; stefano.pro@opbg.net; 6 Neurosurgery Unit, Bambino Gesù Children’s Hospital, IRCCS, 00165 Rome, Italy; franco.randi@opbg.net; 7 Airway Surgery Unit, Pediatric Surgery Department, Bambino Gesù Children’s Hospital, IRCCS, 00165 Rome, Italy; marilena.trozzi@opbg.net (M.T.); duino.meucci@opbg.net (D.M.); 8 Neonatal Intensive Care Unit, Sant’Anna Hospital of Ferrara, 44124 Ferrara, Italy; agostina.solinas@unife.it

**Keywords:** congenital myasthenia, stridor, MUSK, RASPN, DOK7, neonate, next-generation sequencing

## Abstract

Herein, we present a newborn female with congenital vocal cord paralysis who required a tracheostomy in the neonatal period. She also presented with feeding difficulties. She was later diagnosed with a clinical picture of congenital myasthenia, associated with three variants of the MUSK gene: the 27-month follow-up was described. In particular, the c.565C>T variant is novel and has never been described in the literature; it causes the insertion of a premature stop codon (p.Arg189Ter) likely leading to a consequent formation of a truncated nonfunctioning protein. We also systematically collected and summarized information on patients’ characteristics of previous cases of congenital myasthenia with neonatal onset reported in the literature to date, and we compared them to our case. The literature reported 155 neonatal cases before our case, from 1980 to March 2022. Of 156 neonates with CMS, nine (5.8%) had vocal cord paralysis, whereas 111 (71.2%) had feeding difficulties. Ocular features were evident in 99 infants (63.5%), whereas facial-bulbar symptoms were found in 115 infants (73.7%). In one hundred sixteen infants (74.4%), limbs were involved. Respiratory problems were displayed by 97 infants (62.2%). The combination of congenital stridor, particularly in the presence of an apparently idiopathic bilateral vocal cord paralysis, and poor coordination between sucking and swallowing may indicate an underlying congenital myasthenic syndrome (CMS). Therefore, we suggest testing infants with vocal cord paralysis and feeding difficulties for MUSK and related genes to avoid a late diagnosis of CMS and improve outcomes.

## 1. Introduction

Congenital myasthenia syndromes (CMS) are rare but often treatable disorders characterized by fatigable muscle weakness and associated with incorrect signal transmission at the motor endplate (EP) that result from defects in single or multiple proteins. Initially, CMS were classified according to the location of the mutated protein as presynaptic, synaptic basal lamina-associated, and postsynaptic. The present classification involves CMS due to defects in protein glycosylation, where the abnormal proteins are located anywhere in the EP, and other causes of neurotransmission deficiency [1].

Only in recent years, as the genetic knowledge of CMS continues to grow, have disease-associated mutations been discovered in novel genes producing neuromuscular junction proteins, expanding the spectrum of this disorder [2].

MUSK (Muscle Specific Receptor Tyrosine Kinase) is a protein-coding gene located on human chromosome 9q31.3–q32. Mutations of this gene are associated with Fetal Akinesia Deformation Sequence (FADS) [3] and Congenital Myasthenic Syndrome 9, associated with Acetylcholine Receptor Deficiency [4]. Indeed, muscle-specific receptor tyrosine kinase plays a central role in the expression and aggregation of acetylcholine receptors at the neuromuscular junction (NMJ) level, the synapse between the motor neuron and the skeletal muscle [5].

Herein, we present a newborn female with congenital vocal cord paralysis who required a tracheostomy in the neonatal period. She also presented with feeding difficulties. She was later diagnosed with a clinical picture of congenital myasthenia, associated with three variants of the MUSK gene. The aim of this manuscript was to describe her follow-up and compare this case to previously reported CMS cases with neonatal onset.

## 2. Materials and Methods

### 2.1. Lung Function Tests

Lung function was assessed using analysis of the tidal-volume and flow-volume loop using an ultrasonic flowmeter (ndd Medical Technologies, Zurich, Switzerland) connected to an Exhalyzer D (Eco Medics, Dürnten, Switzerland). Neonatal lung function tests (LFTs) were performed in a neutral supine position during normal sleep, without any sedation, recording at minimum 10 consecutive breaths of each patient while also measuring the following parameters: tidal volume (Vt, mL/kg), respiratory rate (RR), and the ratio of time to reach peak tidal expiratory flow over total expiratory time (tPTEF/tE), as previously described [6].

### 2.2. Genetic Analysis

Genomic DNA was extracted from circulating leukocytes collected from the proband. Next-generation sequencing (NGS) targeted at genes causing congenital neuropathies/myopathies was performed using the Trusight ONE kit for clinical exome (Illumina). DNA capture, enrichment, and paired-end sequencing with a read length of 149 bp were performed using the Illumina NextSeq 550 platform with a sequencing depth of 100X. The Illumina VariantStudio 3.0 data analysis software was used to annotate the variants. Conventional Sanger sequencing was performed using ABI 3130xl capillary sequencer (Applied Biosystem) to confirm the variants identified by NGS in the proband and in her parents.

### 2.3. Neurophysiology Tests

Intraoperative laryngeal electromyography (LEMG) was performed using laryngeal reflex (LAR), with transcranial motor evoked potentials (tcMEP) directly applied to the bilateral posterior cricoarytenoid and thyroarytenoid muscles. Brainstem Auditory Evoked Potentials (BAEPs), electroneurography (ENG), electromyography (EMG), and ulnar repetitive nerve stimulation (RNS) at 3–30 Hz were performed according to standardized protocols [7,8,9].

### 2.4. Review of the Literature of Neonatal-Onset Congenital Myasthenia

In order to review the literature about the neonatal onset of CMS, an extensive literature search in the MEDLINE database (via PubMed) has been performed up to 14 March 2022. The following keywords “neonatal onset congenital myasthenia” OR “congenital myasthenia” AND “neonate” were searched as entry terms as well. All 252 retrieved articles were screened, and then full texts of records deemed eligible for inclusion were assessed. References in the relevant papers were also reviewed, and further articles were added if necessary. Papers written in languages other than English were excluded. Information on patients’ characteristics, with age at onset, clinical forms (ocular/facial-bulbar/limb/respiratory), vocal cord paralysis, feeding difficulties, and genetic diagnosis were systematically collected and compared to our case. Parents signed a written informed consent regarding publishing data of their infant.

## 3. Case Report

### 3.1. Clinical Report during NICU Stay

A female infant was born at 38 weeks gestation to a 35-year-old primigravida via elective caesarean section (because of previous retinal detachment). The parents reported no miscarriages, neurological disorders, or autoimmune diseases in the family history. The birthweight was 2845 g (AGA, Z-score: −0.48 SDS), the length was 52 cm (Z-score: 1.98 SDS), and the head circumference was 34.5 cm (Z-score: 0.82 SDS). At birth, she presented hypotonia, cyanosis, and stridor, with worsening respiratory distress, requiring nasotracheal intubation and mechanical ventilation. The Apgar scores at 1, 5, and 10 min were 4, 6, and 8, respectively. A bilateral vocal cord palsy in the adduction was noted. No dysmorphic features were noted.

At 8 days of life, she was referred under mechanical ventilation to our III-level pediatric hospital for further examinations. A laryngo-tracheo-bronchoscopy revealed uncoordinated vocal fold movements, and she could be extubated in spontaneous breathing. LFTs revealed a decreased respiratory flow, especially during the inspiratory phase.

Her brainstem auditory evoked potentials (BAEPs) were within normal limits.

Therefore, the decision was to perform an Endoscopic Arytenoid Latero-Abduction (EALA) [10,11]. However, due to the persistence of stridor and progressive respiratory distress, requiring noninvasive respiratory support, she underwent a tracheostomy at 46 days of life.

Intraoperative LEMG showed reproducibility of motor evoked potentials by a train of 8 stimuli at 250 Hz at a threshold of 300mA and an activation of the chordal structures by direct stimulation of the muscles explored using a train of 5 stimuli at 250 Hz, with an activation threshold at 25 mA. No spontaneous neurotonic activity during LEMG was noted, while asynchronous activity was detected during spontaneous respiratory activity, better identified in the left muscles.

The infant quickly improved without requiring respiratory support yet 48 h after surgery. She had feeding difficulties with good sucking but poor swallow, consequent breastfeeding failure, and need for nasogastric tube (NGT) feeding for the first weeks of life. Enteral nutrition was always tolerated, with normal upper gastrointestinal contrast-enhanced study, gastric emptying scintigraphy, and multichannel intraluminal impedance-pH monitoring.

An automated auditory brainstem response (AABR) test revealed a bilateral normal response.

At 3 months of life, she was discharged home and able to be fed via a feeding bottle.

### 3.2. Genetic Analysis

First-level genetic analysis revealed a normal karyotype and a normal SNP array. NGS detected the presence of the following variants in the MUSK gene (NM_005592.3): c.565C>T in exon 5, and c.2287G>A and c.2368G>A in exon 15, leading to the diagnosis of CMS. The c.565C>T and c.2287G>A were inherited from the mother and the c.2368G>A was inherited from the father.

The novel c.565C>T variant has never been described in the literature or reported in public reference databases (i.e., Genome Aggregation Database: accessible on https://gnomad.broadinstitute.org/ (accessed on 5 January 2023); dbSNP: accessible on https://www.ncbi.nlm.nih.gov/snp/ (accessed on 5 January 2023); it causes the insertion of a premature stop codon (p.Arg189Ter) likely leading to a consequent formation of a truncated nonfunctioning protein.

The c.2287G>A variant, which causes the aminoacidic change p.Ala763Thr (rs199507468), is reported as a “variant of uncertain significance” (VUS, ClinVar Variation ID:839724), while the c.2368G>A variant, leading to the missense change p.Val790Met (rs199476083), is reported as pathogenic (ClinVar Variation ID:8239).

### 3.3. Follow-Up

At 8 months of life, a laryngo-tracheo-bronchoscopy was repeated, with the finding of laryngeal dyskinesia and moderate tracheomalacia. Intraoperative LEMG showed the absence of spontaneous neurotonic activity, a reduced threshold of stimulation of motor evoked potentials, and the absence of a laryngeal reflex.

At 10 months of life, ENG and RNS were normal. EMG revealed a myopathic pattern with a small amplitude and short duration polyphasic motor unit action potentials (MUAPs), especially from an upper limbs examination.

The last polysomnography was performed at the age of 21 months in spontaneous breathing, showing normal O_2_ and CO_2_ levels. Salbutamol therapy was started at the lowest dosage, given the young age of the patient, at the age of 15 months. Currently, she is taking 0.4 mg twice a day, the treatment is well tolerated, and the baby showed significant motor improvement.

At the time of writing, the baby is at 27 months of life. She has normal ocular motility with mild bilateral ptosis in the context of mild facial hypomimia. She’s still a carrier of tracheostomy, but she is on spontaneous breathing even at night. She has good head and trunk control.

She is able to maintain an upright position even without support, she gets up from the squatted position without upper limb support, and she takes a few steps with the support of the upper limbs. Osteotendinous reflexes are normally evoked in the four limbs. She has a ligamentous hyperlaxity. She regularly follows motor physiotherapy sessions for the motor delay. Regular cardiac assessments are being performed. The baby has started to pronounce some words.

## 4. Results

In Table 1 we summarized cases of congenital myasthenic syndrome with neonatal onset previously described and compared them to our case. The literature reported one hundred fifty-five neonatal cases before our case, from 1980 to March 2022 [4,12,13,14,15,16,17,18,19,20,21,22,23,24,25,26,27,28,29,30,31,32,33,34,35,36,37,38,39,40,41,42,43,44,45,46,47,48,49,50,51,52,53,54,55,56,57,58,59,60]. Therefore, of 156 neonates with congenital myasthenic syndrome, nine (5.8%) had vocal cord paralysis, whereas 111 (71.2%) had feeding difficulties. Seventy-seven/135 infants (57.0%) were males. Ocular features were evident in 99 infants (63.5%), whereas facial-bulbar symptoms were found in 115 infants (73.7%). In one hundred sixteen infants (74.4%), limbs were involved. Respiratory problems were displayed by 97 infants (62.2%).

The genetic diagnosis was available in 137 patients (87.8%). Among these 137 patients, the most involved gene was RASPN in 24 cases (17.5%), followed by COL13A1 in 15 cases (10.9%), CHAT in 14 cases (10.2%), DOK7 in 14 cases (10.2%), COLQ in 13 cases (9.5%), SLC5A7 in 12 cases (8.8%), MUSK in nine cases (6.6%), CHRNE in nine cases (6.6%), ACHR in eight cases (5.8%), CHRND in six cases (4.4%), CHRNB1 in five cases (3.6%), PREPL in three cases (2.2%), GFPT1 in two cases (1.5%), ALG2 in a case (0.7%), DMD in a case (0.7%) and SLC18A3 in the remaining case (0.7%). In Table 2 we reported clinical features according to the involved gene.

## 5. Discussion

Herein, we report the case of an infant presenting with congenital vocal cord paralysis (requiring a tracheostomy) and feeding difficulties in the neonatal period. These features led us to the diagnosis of CMS. Indeed, we initially found EMG myopathic alteration (as reported previously) [61,62,63]. RNS was normal but it was performed only from the distal muscle; it was not possible to carry out from the proximal muscle (in the literature are reported decremental responses from proximal muscles) [63]. These findings led us to investigate the child with a next-generation sequencing approach, targeted to genes causing congenital neuropathies/myopathies: we identified three variants of the MUSK gene.

MUSK (OMIM #601296) is a gene encoding for a receptor tyrosine kinase required to form and maintain the neuromuscular junction. Shigemoto et al. presented in 2008 the first evidence that muscle-specific kinase (MuSK) antigen can cause myasthenia in animals [64].

MuSK regulates presynaptic differentiation by generating the clustering of Lrp4, which operates as a direct retrograde signal for presynaptic differentiation. Neuronal Agrin, which is produced by motor nerve terminals and binds to Lrp4, a member of the low-density lipoprotein receptor family, stabilizes developing synapses by encouraging additional interaction between Lrp4 and MuSK and enhancing MuSK kinase activity. Moreover, an inside-out ligand, docking protein-7 (Dok-7), which is recruited to tyrosine-phosphorylated MuSK and boosts MuSK kinase activity, stimulates MuSK phosphorylation. Mutations in MUSK and genes that act in the MuSK signaling system (including DOK7) induce congenital myasthenia [65].

In recent years, several case reports or case series have described the complex clinical features associated with congenital myasthenic syndrome in neonatal age, as summarized in Table 1. Different congenital myasthenia-related genes have been described, encoding for the enzyme acetylcholine esterase, nicotinic acetylcholine receptors, acetylcholine transporters, choline uptake transporters, oligopeptidases involved in the trafficking of vesicular Ach transporter, collagen Q (which anchors acetylcholine esterase to the basal lamina), downstream of kinase 7 (a cytoplasmic adaptor of MusK), and proteins involved in the formation and maintenance of the neuromuscular synapse (independently of the acetylcholine receptor clustering pathway such as in the case of COL13A1) [1,51,66,67].

From the review of the literature, it appears that the phenotypic spectrum associated with MUSK variants is variable and comprises different features [1,49,53,59]. All infants with MUSK mutations had respiratory symptoms, whereas ocular, facial-bulbar, and limb features were reported in most patients, as in our infant with three MUSK variants.

The first c.565C>T variant is novel and has never been described in the literature; it causes the insertion of a premature stop codon (p.Arg189Ter), likely leading to a consequent formation of a truncated nonfunctioning protein.

The second variant, c.2287G>A, in cis with the c.565C>T, causes the missense substitution p.Ala763Thr and is classified in ClinVar database as VUS; however, it is already described in compound heterozygosity with a truncating variant in MUSK gene in two siblings with neonatal respiratory failure secondary to isolated vocal cord paralysis (requiring tracheostomy in one of them), failure to thrive and feeding intolerance [49].

The third c.2368G>A variant that causes the aminoacidic change p.Val790Met has already been reported as pathogenic and it has been described in the literature as associated with congenital myasthenic syndromes [61,68,69].

Our case further supports that laryngeal stridor, vocal cord paralysis, and feeding difficulties could be the early diagnostic clues of a congenital myasthenic syndrome with neonatal onset due to a mutation in the MUSK gene. Previously, Jephson et al. reported six patients with DOK7 mutations presenting congenital stridor and feeding difficulties. Despite all six children having had neonatal symptoms, the mean age at CMS diagnosis was 5 years and 9 months in this cohort [30].

Next-generation sequencing will continue recognizing newer CMS genes, enhancing an earlier diagnosis, and expanding the spectrum of current phenotypes. Furthermore, early recognition of these disorders is crucial, considering they usually respond favorably to drugs enhancing neuromuscular transmission [1]. The choice of medication varies with the CMS subtype, and genetic testing can help guide management. Although the majority of individuals with CMS benefit from AChE inhibitors (pyridostigmine), some myasthenic symptoms may remain refractory to treatment. Beta-2-agonists have been described as effective in several CMS subtypes (in particular in endplate AChE deficiency and in patients with DOK7 pathogenic variants). Moreover, in patients with CMS responsive to AChE inhibitors, it may mitigate the detrimental effects on the endplate fine structure caused by long-term anticholinesterase treatment [70].

Concerning patients with MUSK variants, salbutamol has been described as an effective and first-line therapy [67], but the mechanisms by which open-channel blockers improve neuromuscular transmission are still not understood [66]. In our case, salbutamol treatment was started at the age of 15 months and is well tolerated to date. Along with response to treatment, we hope to propose to our patient and her family the opportunity of decannulation as soon as possible.

The main limitation of data available in the literature is the lack of a genetic diagnosis in all cases of CMS with a neonatal onset, further reducing the sample of infants with MUSK variants. Furthermore, the treatment of previously reported cases was not described in all CMS patients with neonatal onset, due to a short follow-up. Therefore, this case with a 27-month follow-up, reporting a novel pathogenic variant associated with CMS in neonatal age and the response to the treatment with salbutamol, is particularly noteworthy. We suggest testing infants with vocal cord paralysis and feeding difficulties for MUSK and related CMS genes to avoid a late diagnosis and improve outcomes, given the possibility of a target treatment.

## 6. Conclusions

The combination of congenital stridor, particularly in the presence of an apparently idiopathic bilateral vocal cord paralysis, and poor coordination between sucking and swallowing may indicate an underlying CMS. These infants should be referred to III-level centers for neurophysiology and genetic tests earlier as possible because CMS represents a rare but treatable cause of early-onset muscle weakness, such as in the case of CMS due to MUSK variants.

## Figures and Tables

**Table 1:** Cases of congenital myasthenic syndrome with neonatal onset reported in the literature. A: Ambenomium; AChEI: Acetylcholinesterase Inhibitor; Alb: Albuterol; DAP: Diaminopyridine; E: Ephedrine; EALA: Endoscopic Arytenoid Latero-Abduction; F: Fluoxetine; N: Neostigmine; N/A: Not available; P: Pyridostigmine; PF: Plasmaferesis; S: Steroids; SA: Salbutamol.

	First Author, Year	Sex	Onset	Clinical Forms (Ocular/Facial-Bulbar/Limb/ Respiratory) (+ Present, − Absent)	Vocal Cord Paralysis or Stridor	Feeding Difficulties	Gene Involved	Medical Treatment (+ Response, +/− Partial Response, or − No Response)	Surgical Treatments
1	Smit, 1980 [12]	M	At birth	−/+/+/+	+	−	N/A	P+	N/A
2	Hageman, 1986 [13]	M	At birth	−/+/+/−	−	+	N/A	P+	Gastrostomy, Tracheostomy
3	Roach, 1986 [14]	M	At birth	+/+/−/+	−	+	N/A	P+	N/A
4	Roach, 1986 [14]	M	At birth	+/+/−/+	−	−	N/A	P+	N/A
5	Roach, 1986 [14]	M	At birth	−/−/−/+	−	−	N/A	P−, S−, PF−	N/A
6	Engel, 1990 [15]	F	At birth	−/+/+/−	−	+	N/A	P−	N/A
7	Engel, 1990 [15]	F	At birth	+/+/+/+	−	−	N/A	P+	N/A
8	Engel, 1990 [15]	F	At birth	+/+/−/+	−	+	N/A	P+	N/A
9	Vial, 1991 [16]	M	At birth	+/−/−/−	−	−	N/A	N/A	N/A
10	Vial, 1991 [16]	M	At birth	+/−/−/−	−	−	N/A	N/A	N/A
11	Vial, 1991 [16]	F	At birth	+/+/+/+	−	+	N/A	N/A	N/A
12	Ohno, 1997 [17]	M	At birth	+/+/−/−	−	+	CHRNE	P+/−	N/A
13	Quiram, 1999 [18]	F	At birth	−/+/−/+	−	+	CHRNB1	N/A	Gastrostomy
14	Mullaney, 2000 [19]	M	At birth	−/+/+/−	−	−	N/A	P+, S+	N/A
15	Brownlow, 2001 [20]	M	At birth	−/+/+/+	−	+	CHRND	P-	N/A
16	Zafeiriou, 2003 [21]	F	At birth	+/+/−/−	−	−	CHRND	P+	N/A
17	Zafeiriou, 2003 [21]	F	At birth	−/+/−/−	−	+	N/A	P+	N/A
18	Zafeiriou, 2003 [21]	F	At birth	+/+/+/+	−	−	N/A	N/A	N/A
19	Zafeiriou, 2003 [21]	M	At birth	−/+/+/−	−	−	N/A	P+	Tracheostomy
20	Zafeiriou, 2003 [21]	M	At birth	+/+/−/−	−	+	N/A	N/A	N/A
21	Zafeiriou, 2003 [21]	F	At birth	+/+/+/+	−	−	N/A	P+	N/A
22	Zafeiriou, 2003 [21]	F	At birth	+/+/+/+	−	−	N/A	P+	N/A
23	Zafeiriou, 2003 [21]	M	At birth	+/+/+/−	−	−	N/A	P−	N/A
24	Ioos, 2004 [22]	F	At birth	−/+/+/+	−	+	RAPSN	A+	Tracheostomy
25	Ioos, 2004 [22]	M	At birth	−/−/−/+	−	−	RAPSN	P+	N/A
26	Ioos, 2004 [22]	M	At birth	−/+/−/+	−	+	RAPSN	P+	Tracheostomy
27	Barisic, 2005 [23]	F	At birth	+/−/−/+	−	−	CHAT	P−	N/A
28	Barisic, 2005 [23]	F	At birth	+/−/+/+	−	−	CHAT	P+	N/A
29	Muller, 2006 [24]	M	At birth	+/+/+/−	−	+	CHRND	P+	N/A
30	Mihaylova, 2008 [25]	F	At birth	+/+/+/+	−	+	COLQ	P+	N/A
31	Mihaylova, 2008 [25]	F	At birth	+/+/+/+	−	+	COLQ	P+	N/A
32	Mihaylova, 2008 [25]	M	At birth	+/−/+/+	−	+	COLQ	P+	N/A
33	Mihaylova, 2008 [25]	F	At birth	+/−/+/+	−	−	COLQ	P+	N/A
34	Mihaylova, 2008 [25]	M	At birth	+/−/+/+	−	−	COLQ	None	N/A
35	Mihaylova, 2008 [25]	M	At birth	+/+/−/−	−	+	COLQ	P+	N/A
36	Mihaylova, 2008 [25]	M	At birth	+/−/+/+	−	−	COLQ	P+/−	N/A
37	Mihaylova, 2008 [25]	F	At birth	+/−/+/+	−	−	COLQ	P+/−	N/A
38	Mihaylova, 2008 [25]	F	At birth	+/−/+/+	−	−	COLQ	P+	N/A
39	Mihaylova, 2008 [25]	M	At birth	+/−/+/+	−	−	COLQ	P+	N/A
40	Mihaylova, 2008 [25]	M	At birth	+/+/+/+	−	+	COLQ	None	N/A
41	Faber, 2009 [26]	M	At birth	+/−/−/−	−	−	ACHR	N/A	N/A
42	Faber, 2009 [26]	F	At birth	+/−/+/−	−	−	ACHR	N +	N/A
43	Faber, 2009 [26]	M	At birth	−/+/−/−	−	+	ACHR	N +	N/A
44	Faber, 2009 [26]	M	At birth	+/+/−/−	−	+	ACHR	N/A	N/A
45	Mallory, 2009 [27]	F	At birth	−/−/−/+	−	−	CHAT	P+	Gastrostomy
46	Yeung, 2009 [28]	F	At birth	+/−/−/+	−	+	CHAT	P+	Gastrostomy
47	Ben Ammar, 2010 [29]	F	At birth	+/+/+/−	−	−	DOK7	P−	N/A
48	Ben Ammar, 2010 [29]	M	Antenatal	+/+/+/+	−	+	DOK7	P−, DAP+	N/A
49	Ben Ammar, 2010 [29]	F	Antenatal	+/+/+/−	−	−	DOK7	P−, DAP+	N/A
50	Ben Ammar, 2010 [29]	M	At Birth	−/+/+/−	−	−	DOK7	N/A	N/A
51	Ben Ammar, 2010 [29]	F	At birth	−/+/+/+	+	+	DOK7	P−, DAP+	N/A
52	Ben Ammar, 2010 [29]	F	At birth	+/+/−/+	+	−	DOK7	P−, DAP+	N/A
53	Jephson, 2010 [30]	N/A	At birth	−/−/−/+	+	+	DOK7	N/A	Tracheostomy and gastrostomy
54	Jephson, 2010 [30]	N/A	At birth	−/−/−/+	+	+	DOK7	N/A	N/A
55	Jephson, 2010 [30]	N/A	At birth	−/−/−/+	+	+	DOK7	N/A	Cordotomy and aryepiglottoplasty
56	Jephson, 2010 [30]	N/A	At birth	−/−/−/+	−	+	DOK7	N/A	Gastrostomy
57	Jephson, 2010 [30]	N/A	At birth	−/−/−/+	−	+	DOK7	N/A	N/A
58	Jephson, 2010 [30]	N/A	At birth	−/−/−/+	+	+	DOK7	N/A	Tracheostomy
59	Schara, 2010 [31]	M	At birth	+/+/+/+	−	−	CHAT	P+, DAP+	Tracheostomy
60	Schara, 2010 [31]	M	At birth	+/+/+/+	−	+	CHAT	P+	N/A
61	Schara, 2010 [31]	M	At birth	+/+/+/−	−	−	CHAT	P+	N/A
62	Schara, 2010 [31]	M	At birth	+/−/+/+	−	−	CHAT	P+	Tracheostomy
63	Das, 2014 [32]	M	At birth	−/−/+/+	−	+	RAPSN	P+	Gastrostomy and Nissen
64	Dilena, 2014 [33]	M	At birth	+/+/+/+	−	+	CHAT	P+; DAP+	Tracheostomy and gastrostomy
65	Webster, 2014 [34]	M	At birth	+/+/+/+	−	+	CHRNE	P+; SA+	N/A
66	Guo, 2015 [35]	M	At birth	−/−/−/+	+	+	DMD	N/A	N/A
67	Bauchè, 2016 [36]	M	At birth	−/+/+/−	−	+	SLC5A7	AChEI+	N/A
68	Bauchè, 2016 [36]	F	At birth	−/+/−/+	−	−	SLC5A7	AChEI+	Tracheostomy
69	Bauchè, 2016 [36]	M	At birth	−/+/−/−	−	+	SLC5A7	AChEI−, SA−	N/A
70	Bauchè, 2016 [36]	M	At birth	−/+/+/+	−	−	SLC5A7	AChEI+	N/A
71	Natera-de Benito, 2016 [37]	F	At birth	−/+/+/+	−	+	RAPSN	P+	N/A
72	Natera-de Benito, 2016 [37]	F	Prenatal/at birth	−/+/+/+	−	+	RAPSN	P+	N/A
73	Natera-de Benito, 2016 [37]	M	Prenatal/at birth	−/+/+/+	−	+	RAPSN	P+	N/A
74	Natera-de Benito, 2016 [37]	M	Prenatal/at birth	−/+/+/−	−	+	RAPSN	P+	N/A
75	Natera-de Benito, 2016 [37]	F	Prenatal/at birth	−/+/+/−	−	+	RAPSN	N/A	N/A
76	Natera-de Benito, 2016 [37]	M	At birth	−/+/+/+	−	+	RAPSN	P+, DAP+	N/A
77	Natera-de Benito, 2016 [37]	M	At birth	−/+/+/−	−	+	RAPSN	P+	N/A
78	Natera-de Benito, 2016 [37]	M	At birth	−/+/+/−	−	+	RAPSN	P+, DAP+	N/A
79	Natera-de Benito, 2016 [37]	M	At birth	−/+/+/+	−	+	RAPSN	P+	N/A
80	Natera-de Benito, 2016 [37]	F	At birth	−/+/+/+	−	+	RAPSN	P+	N/A
81	Natera-de Benito, 2016 [38]	F	Neonatal period	+/+/+/+	−	+	CHRNE	P−	N/A
82	Natera-de Benito, 2016 [38]	M	Neonatal period	+/+/−/+	−	+	CHRNE	P+	N/A
83	Natera-de Benito, 2016 [38]	M	Neonatal period	+/+/+/+	−	+	CHRNE	P−, DAP−	N/A
84	Natera-de Benito, 2016 [38]	F	Neonatal period	+/+/+/+	−	+	CHRNE	P−, DAP−	N/A
85	Shen, 2016 [39]	F	At birth	+/+/+/+	−	+	ACHR	P+, DAP+	Gastrostomy
86	Shen, 2016 [40]	M	At birth	+/+/+/+	−	N/A	ACHR	P−, Quinidine solfate (N/A)	N/A
87	Shen, 2016 [40]	F	At birth	−/−/+/−	−	N/A	ACHR	P+/−	N/A
88	Shen, 2016 [40]	F	At birth	−/−/+/−	−	N/A	ACHR	N/A	N/A
89	Bhoopalan, 2017 [41]	M	At birth	+/+/+/−	−	+	DOK7	Alb +	N/A
90	Winters, 2017 [42]	M	Prenatal	−/+/+/−	−	+	RASPN	N/A	N/A
91	Banerjee, 2018 [43]	F	Prenatal	−/−/+/−	−	+	SLC5A7	P−, DAP−, F−, SA − (died)	N/A
92	Banerjee, 2018 [43]	M	Prenatal	−/−/+/−	−	+	SLC5A7	P − (died)	N/A
93	Liu, 2018 [44]	M	At birth	+/+/+/+	−	+	CHAT	None (died)	N/A
94	Pardal-Fernandez, 2018 [45]	M	Prenatal, at birth	−/−/+/+	−	+	SCL5A7	P+/−	N/A
95	Silva, 2018 [46]	F	At birth	+/+/+/−	−	+	PREPL	N/A	N/A
96	Espinoza, 2019 [47]	M	At birth	+/+/+/+	−	+	RAPSN	P+	N/A
97	Helman, 2019 [48]	M	Prenatal, At birth	−/−/+/−	−	−	GFPT1	N/A	N/A
98	Helman, 2019 [48]	M	At birth	−/−/+/+	−	−	GFPT1	N/A	N/A
99	Murali, 2019 [49]	M	At birth	−/−/−/+	+	−	MUSK	None	Tracheostomy
100	Murali, 2019 [49]	M	At birth	−/−/−/+	+	−	MUSK	None	Tracheostomy
101	Rodríguez Cruz, 2019 [50]	M	At birth	−/−/+/+	−	+	SLC5A7	P+, SA (N/A)	N/A
102	Rodríguez Cruz, 2019 [50]	M	1 month	−/−/−/+	−	+	SLC5A7	P−, SA+	N/A
103	Rodríguez Cruz, 2019 [50]	F	At birth	+/−/+/−	−	+	SLC5A7	P+, SA+	N/A
104	Rodríguez Cruz, 2019 [50]	M	At birth	−/+/+/+	−	+	SLC5A7	P−, DAP−, SA− (died)	N/A
105	Rodríguez Cruz, 2019 [50]	M	At birth	−/+/+/−	−	+	SLC5A7	P−; (died)	Tracheostomy
106	Rodríguez Cruz, 2019 [51]	F	At birth	+/+/+/+	+	+	MUSK	P−	Tracheostomy and gastrostomy
107	Rodríguez Cruz, 2019 [51]	F	At birth	+/+/+/+	−	+	COL13A1	P−, DAP+, SA+	Gastrostomy
108	Rodríguez Cruz, 2019 [51]	M	At birth	+/+/−/−	−	+	COL13A1	None	N/A
109	Rodríguez Cruz, 2019 [51]	F	At birth	+/+/+/+	−	+	COL13A1	P−	N/A
110	Rodríguez Cruz, 2019 [51]	M	At birth	+/+/+/−	−	+	COL13A1	P−, SA+	N/A
111	Rodríguez Cruz, 2019 [51]	M	At birth	+/+/+/+	−	+	COL13A1	DAP+, SA+	N/A
112	Rodríguez Cruz, 2019 [51]	F	At birth	+/+/+/+	−	+	COL13A1	P+, SA	N/A
113	Rodríguez Cruz, 2019 [51]	F	At birth	+/+/+/−	−	+	COL13A1	None	N/A
114	Rodríguez Cruz, 2019 [51]	M	At birth	+/+/+/+	−	+	COL13A1	DAP+, SA+	Tracheostomy and gastrostomy
115	Rodríguez Cruz, 2019 [51]	M	At birth	+/+/+/+	−	+	COL13A1	DAP+, SA+	N/A
116	Rodríguez Cruz, 2019 [51]	M	At birth	+/+/+/+	−	+	COL13A1	P+, SA+	N/A
117	Rodríguez Cruz, 2019 [51]	M	At birth	+/+/−/+	−	+	COL13A1	P−	N/A
118	Rodríguez Cruz, 2019 [51]	F	At birth	+/+/−/−	−	+	COL13A1	P−	N/A
119	Rodríguez Cruz, 2019 [51]	F	At birth	+/+/−/−	−	−	COL13A1	P−	N/A
120	Rodríguez Cruz, 2019 [51]	M	At birth	+/+/+/−	−	+	COL13A1	None	N/A
121	Rodríguez Cruz, 2019 [51]	F	At birth	+/+/+/+		+	COL13A1	DAP+, SA+	Tracheostomy and gastrostomy
122	Bonanno, 2020 [52]	F	At birth	+/+/+/+	−	+	CHRND	P− (died at 3 months)	N/A
123	Bonanno, 2020 [52]	F	At birth	+/+/+/+	−	+	CHRND	P−, SA − (died at 4 months)	N/A
124	Della Marina, 2020 [53]	N/A	At birth	+/+/+/−	−	+	CHAT	P (muscular weakness during follow-up)	N/A
125	Della Marina, 2020 [53]	N/A	Prenatal (reduced fetal movements/At birth	+/+/+/−	−	+	CHAT	P (muscular weakness during follow-up)	N/A
126	Della Marina, 2020 [53]	N/A	Prenatal (reduced fetal movements)/At birth	+/+/+/−	−	+	CHAT	P (muscular weakness during follow-up)	N/A
127	Della Marina, 2020 [53]	N/A	At birth	+/+/+/−	−	+	COLQ	E+	N/A
128	Della Marina, 2020 [53]	N/A	At birth	+/+/+/−	−	+	COLQ	E+	N/A
129	Della Marina, 2020 [53]	N/A	Prenatal (reduced fetal movements)/At birth	+/+/+/−	−	−	CHRNE	P+	N/A
130	Della Marina, 2020 [53]	N/A	At birth	+/+/+/−	−	−	CHRNE	P+	N/A
131	Della Marina, 2020 [53]	N/A	At birth	+/+/+/−	−	−	CHRNE	P+	N/A
132	Della Marina, 2020 [53]	M	At birth	+/+/+/+	−	+	CHRND	P+	N/A
133	Della Marina, 2020 [53]	F	At birth	+/+/+/+	−	+	CHRNB1	P+	N/A
134	Della Marina, 2020 [53]	M	At birth	+/+/+/+	−	+	MUSK	P+/−, E+	Tracheostomy
135	Della Marina, 2020 [53]	N/A	At birth	+/+/+/+	−	+	RAPSN	P+	N/A
136	Della Marina, 2020 [53]	N/A	At birth	+/+/+/+	−	+	RAPSN	P+	N/A
137	Della Marina, 2020 [53]	N/A	At birth	+/+/+/+	−	+	RAPSN	P+	N/A
138	Della Marina, 2020 [53]	N/A	At birth	+/+/+/−	−	+	RAPSN	P+	N/A
139	Della Marina, 2020 [53]	N/A	At birth	+/+/+/−	−	+	RAPSN	P+	N/A
140	Della Marina, 2020 [53]	N/A	At birth	+/+/+/−	−	+	RAPSN	P+	N/A
141	Della Marina, 2020 [53]	N/A	At birth	+/+/+/−	−	+	RAPSN	P+	N/A
142	Della Marina, 2020 [53]	F	Neonatal period	+/+/+/+	−	+	CHRNB1	P+, DAP+	N/A
143	Della Marina, 2020 [53]	M	Neonatal period	+/+/+/+	−	+	MUSK	P+/−, DAP+/−, E +	Tracheostomy
144	Freed, 2020 [54]	M	At birth	−/−/+/+	−	+	CHRNB1	P+ (died at 56 days)	N/A
145	Freed, 2020 [54]	F	At birth	+/+/+/+	−	+	CHRNB1	P+/−; DAP+	N/A
146	Harrar, 2020 [55]	M	At birth	+/+/+/+	−	+	ChAT	P+	Tracheostomy
147	Shen, 2020 [56]	F	At birth	−/+/+/+	−	+	MUSK	P+ (died at 56 days)	N/A
148	Zhang, 2020 [57]	F	At birth	−/+/+/−	−	+	PREPL	P+	N/A
149	Lamond, 2021 [58]	M	At birth	−/+/−/+	−	+	SLC18A3	P+	N/A
150	Prior, 2021 [59]	F	At birth	+/−/−/−	−	+	DOK7	Alb +	N/A
151	Prior, 2021 [59]	M	At birth	+/+/+/−	−	+	RAPSN	P+	
152	Prior, 2021 [59]	F	At birth	+/+/+/+	−	+	MUSK	Alb (walk with support)	Tracheostomy
153	Prior, 2021 [59]	M	At birth	+/+/+/+	−	+	MUSK	Alb	Tracheostomy
154	Prior, 2021 [59]	F	At birth	+/+/+/−	−	+	PREPL	P+	N/A
155	Ehrstedt, 2022 [60]	F	At birth	−/−/+/+	−	+	ALG2	P−, SA +	N/A
156	De Rose, 2023	F	At birth	+/+/+/+	+	+	MUSK	Salbutamol since 15 months of age	EALA then tracheostomy

**Table 2:** Cases of congenital myasthenic syndrome with neonatal onset reported in the literature.

Patients with Genetic Diagnosis (*n* = 137)	Ocular Features	Facial-Bulbar Features	Limb Features	Respiratory Features	Vocal Cord Paralysis or Stridor	Feeding Difficulties
ACHR (*n* = 8)	5/8 (62.5%)	4/8 (50.0%)	5/8 (6.5%)	1/8 (12.5%)	0	3/5 (60.0%)
ALG2 (*n* = 1)	0	0	1 (100%)	1 (100%)	0	1 (100%)
CHAT (*n* = 14)	13/14 (92.9%)	9/14 (64.3%)	11/14 (78.6%)	10/14 (71.4%)	0	8/14 (57.1%)
CHRNB1 (*n* = 5)	4/5 (80.0%)	5/5 (100%)	4/5 (80.0%)	5/5 (100%)	0	5/5 (100%)
CHRND (*n* = 6)	5/6 (83.3%)	6/6 (100%)	5/6 (83.3%)	4/6 (66.7%)	0	5/6 (83.3%)
CHRNE (*n* = 9)	9/9 (100%)	9/9 (100%)	7/9 (77.8%)	5/9 (55.6%)	0	6/9 (66.7%)
COL13A1 (*n* = 15)	15/15 (100%)	15/15 (100%)	11/15 (73.3%)	9/15 (60.0%)	0	14/15 (93.3%)
COLQ (*n* = 13)	13/13 (100%)	6/13 (46.2%)	12/13 (92.3%)	10/13 (7.7%)	0	7/13 (53.8%)
DMD (*n* = 1)	0	0	0	1 (100%)	0	1 (100%)
DOK7 (*n* = 14)	6/14 (42.9%)	7/14 (50.0%)	6/14 (42.9%)	9/14 (64.3%)	6/14 (42.9%)	10/14 (71.4%)
GFPT1 (*n* = 2)	0	0	2 (100%)	0	0	0
MUSK (*n* = 9)	5/9 (55.6%)	7/9 (77.8%)	7/9 (77.8%)	9/9 (100%)	4/9 (44.4%)	7/9 (77.8%)
PREPL (*n* = 3)	2/3 (66.7%)	3/3 (100%)	3/3 (100%)	0	0	3/3 (100%)
RAPSN (*n* = 24)	9/24 (37.5%)	22/24 (91.7%)	22/24 (91.7%)	14/24 (58.3%)	0	23/24 (95.8%)
SLC18A3 (*n* = 1)	0	1 (100%)	0	1 (100%)	0	1 (100%)
SLC5A7 (*n* = 12)	1/12 (8.3%)	6/12 (50.0%)	9/12 (75.0%)	6/12 (50.0%)	0	10/12 (83.3%)

## Data Availability

All data considered for this case report have been included in this article. Articles considered for the review of literature are already available on PubMed.
